# Revision of reversed shoulder arthroplasty: Is a reoperation possible?

**DOI:** 10.1007/s11678-017-0400-x

**Published:** 2017-03-07

**Authors:** Stijn Casier, Bart Middernacht, Alexander Van Tongel, Lieven De Wilde

**Affiliations:** 1 Department of Orthopaedics, University Hospital of Ghent, Gent, Belgium; 2 Department of Orthopaedics, OLV Hospital, Aalst, Belgium; 3 Department of Orthopaedic Surgery and Traumatology, Ghent University Hospital, De Pintelaan 185, 9000 Gent, Belgium

**Keywords:** Pain, Reoperation, Hemiarthroplasty, Spacer, Range of motion, Schmerz, Reoperation, Hemiprothese, Spacer, Bewegungsumfang

## Abstract

**Introduction:**

As the number of reversed shoulder arthroplasty (RSA) procedures increases, the revision rate will also increase. In case of severe bone insufficiency, instability or infection of the primary RSA, revision to another RSA is preferable but not always possible. Hemiarthroplasty (HA), spacers and resection arthroplasty (RA) have been described in this indication.

**Materials and methods:**

Between 2004 and 2016, 20 shoulders in 19 patients were treated at Ghent University Hospital for failed revision of RSA. Nine received a megahead prosthesis, a spacer was implanted in 6, and 5 underwent RA.

**Results:**

Indications for implantation of a megahead prosthesis were loosening RSA (*n* = 5), infection (*n* = 4), dislocation (*n* = 1) and nerve irritation (*n* = 1). Improvement of range of motion was observed. Anterosuperior migration of the prosthesis was noted in 2 patients. Another 2 patients were ultimately revised to RSA. Seven permanent spacers were implanted for infection, of which 2 remain in place till today. The other 5 were revised to RSA. Of the 5 patients treated with RA, 3 were revised further on to RSA, resulting in pain relief and regain of function.

**Discussion:**

Our study shows that a megahead prosthesis has better functional results than RA, but is inferior to RSA. Due to increasing surgical experience and improving technique, 9 patients could ultimately be reconverted to another RSA. A review of current literature is presented. In HA and RA, the functional results are poor, and pain relief is uncertain. Results of spacers are variable and can be satisfactory. Arthrodesis is a last resort.

**Conclusion:**

In our case series study, a hemiarthroplasty can be performed in case of failure of RSA. However, the results are inferior to another RSA.

## Zusammenfassung

**Einleitung:**

Da die Zahl der inversen Schulterprothesen („reversed shoulder arthroplasty“, RSA) steigt, nimmt auch die Revisionsrate zu. Im Fall einer schweren ossären Insuffizienz, Instabilität oder Infektion der primären RSA ist eine Revision mit Erneuerung der RSA wünschenswert, aber nicht immer möglich. Hemiprothesen (HA), Spacer und Resektionsprothesen (RA) wurden für diese Indikation beschrieben.

**Material und Methoden:**

Zwischen 2004 und 2016 wurden 20 Schultern von 19 Patienten im Ghent University Hospital wegen einer fehlgeschlagenen RSA-Revision behandelt. Neun Patienten erhielten eine Großkopfprothese, bei 6 Patienten wurde ein Spacer implantiert und 5 erhielten eine RA.

**Ergebnisse:**

Indikationen für die Implantation einer Großkopfprothese waren eine gelockerte RSA (*n* = 5), Infektion (*n* = 4), Dislokation (*n* = 1) und Nervenreizung (*n* = 1). Eine Verbesserung des Bewegungsumfangs wurde beobachtet. Eine anterosuperiore Migration der Prothese wurde bei 2 Patienten festgestellt. Bei 2 anderen Patienten wurde schließlich eine RSA-Revision durchgeführt. Sieben permanente Spacer wurden aufgrund einer Infektion implantiert, von denen bis heute 2 verblieben sind. Bei den anderen 5 Patienten wurde eine RSA-Revisionsoperation durchgeführt. Von den 5 Patienten, die eine RA erhalten hatten, wurden 3 mit einer neuen RSA versorgt, was in einer Besserung der Schmerzsymptomatik und Wiederherstellung der Funktion resultierte.

**Diskussion:**

Unsere Studie zeigt, dass eine Großkopfprothese bessere funktionelle Ergebnisse zeigt als eine RA, sie einer RSA jedoch unterlegen ist. Aufgrund zunehmender chirurgischer Erfahrung und verbesserter Technik konnten 9 Patienten schließlich einer erneuten RSA-Operation zugeführt werden. Ein Review der aktuellen Literatur wird vorgestellt. Bei HA und RA sind die funktionellen Ergebnisse schlecht, und die Verbesserung der Schmerzsymptomatik ist ungewiss. Die Ergebnisse der Spacer fallen unterschiedlich aus und können als zufriedenstellend bezeichnet werden. Die Arthrodese ist das Ultima ratio.

**Schlussfolgerung:**

Die vorliegende Fallstudie zeigt, dass im Fall eines RSA-Versagens eine Hemiprothese implantiert werden kann. Jedoch sind die Ergebnisse im Vergleich zu einer neuen RSA unterlegen.

## Introduction

In 2011, 66,485 patients underwent a shoulder arthroplasty procedure in the USA, of which 44.2% received a total shoulder arthroplasty (TSA), 32.6% a reversed shoulder arthroplasty (RSA) and 23.2% a hemiarthroplasty (HA). Since its approval by the FDA (US Food and Drug Administration) in 2004, the number of RSA procedures has increased over tenfold in 7 years, from approximately 2000 interventions in 2004 to 21,692 in 2011 [1, 9].

Also, the indications for the RSA have been expanded: whereas the original goal was to treat pseudoparalysis with cuff tear arthropathy, today, RSA is used in the treatment of massive rotator cuff tears, failed total shoulder arthroplasty or hemiarthroplasty, acute fractures or their sequelae, tumors and rheumatoid arthritis [2]. This spectacular increase, combined with a revision rate of 6–10.1% [2–4] signifies a drastic increase in the number of revisions for RSA. The most important indications for such revisions are instability, humeral complications, infections, glenoidal complications and periprosthetic fractures. [2, 6]. Overall, the clinical results in a revision RSA are inferior to those for a primary RSA. When a revision can end with a new RSA, patients usually have better clinical results than with other revision solutions [2, 5].

In some cases, a new RSA cannot be placed because of insufficient or low quality bone stock, infection or refractory instability [7]. In these cases salvage surgery is required. In literature, various procedures have been described: conversion to hemiarthroplasty (classic or a megahead prosthesis) [2, 4, 6, 7], spacer [5, 8] or resection arthroplasty [2, 4, 5]. The primary goal in all these procedures is to establish a stable fulcrum, which can allow for some shoulder function. Arthrodesis remains a theoretical option after RSA failure, knowing it has been described after failed TSA [10].

The purpose of this study was to retrospectively evaluate the outcome of revision RSA with megahead cuff tear arthropathy prosthesis (DePuy-Synthes), spacers or resection arthroplasty. We hypothesise that the results of these techniques offer adequate pain relief and acceptable shoulder function.

## Materials and methods

This study was set up at the University Hospital of Ghent. Approval was obtained from the Ghent University Hospital (UZ Gent) ethics committee (B670201422180). A consecutive series of all patients with failed reversed total shoulder arthroplasty treated with hemiarthroplasty, spacer or resection arthroplasty between 2004 and 2016 were analysed. The senior author performed the operation in all patients. Indications for revision of failed reversed total shoulder arthroplasty included infections, glenoid loosening, instability, malpositioning and suprascapular nerve irritation. Whenever possible, the prosthetic elements were kept in place, or replaced by new ones when needed. If preservation of the reversed shoulder joint was impossible, a salvage procedure was performed: implantation of a megahead prosthesis (massive glenoid bone loss), a permanent spacer or a resection arthroplasty (infection). We grouped our patients according to their indication and for each group we described the surgical technique and the clinical outcome using the Constant score [23] (preoperative; postoperative at 3 months, 6 months, 1 year and annually thereafter).

Prior to surgery, a CT scan was made to assess bone stock and detect surgical difficulties**.**

In case a megahead prosthesis was placed, the approach as described by Redfern and Wallace [24] was used. The remains of the rotator cuff were identified whenever possible; however the subscapularis could not be identified in any patient.

Postoperative radiographs were made at 1 day, 6 months and 1 year after surgery to evaluate position and to detect signs of loosening.

Between September 2006 and September 2016, 550 patients were treated with a primary RSA in the University Hospital, Ghent, by the senior author (LDW). In case of persistent failure of the RSA 20 shoulders in 19 patients were treated with a salvage procedure: 9 were treated with a megahead prosthesis (patients 1–9, patient 1 on the left side), 6 with a permanent spacer because of persistent infection (patients 10–15), and 5 with a resection arthroplasty (patient 1 on the right side, patients 16–19).

## Results

### Cuff tear arthropathy megahead prosthesis

**Fig. 1:**
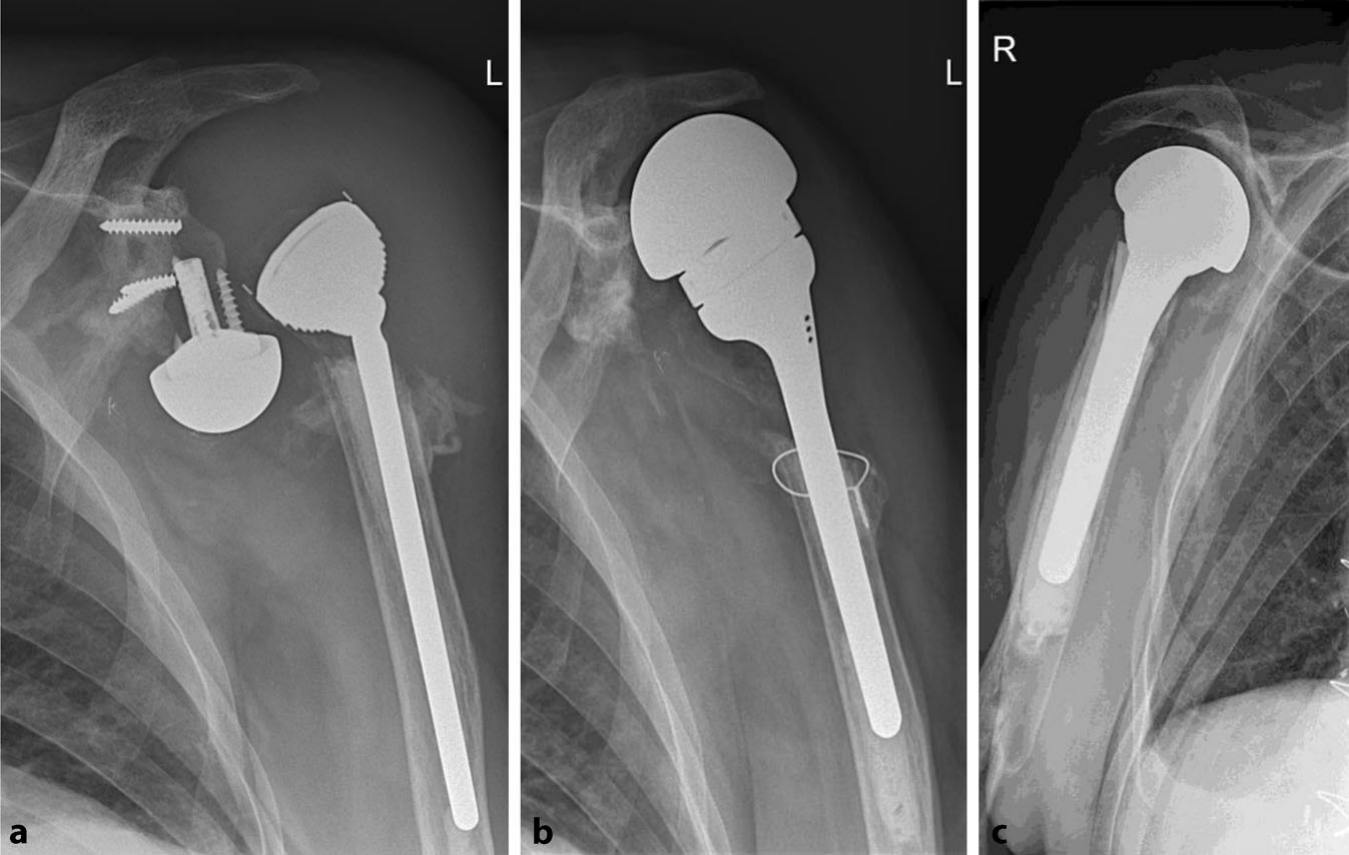
Patient 1: Loosening and luxation of the RSA (*left*), and 2 months after implantation of the megahead prosthesis (*middle*). Attention must be drawn to the fact that the megahead prosthesis is designed with an articular surface larger than other available hemiprostheses (*right*)

Mean age at implantation of the megahead was 75.2 years; 7 of the 9 patients were females. Five of the 9 patients showed clinical and radiographical signs of loosening of the original RSA (patients 1–5; Fig. 1). Four patients presented with infection: patient 4 (see below), patient 5 had an infected fistula, and in patients 6 and 7 cultures confirmed the clinical suspicion. Dislocation (patient 8) and irritation of the suprascapular nerve by one of the screws (patient 9) were other indications. The clinical results are shown in Table 1, along with the degree of retroversion by which the prosthesis was implanted.

**Table 1:** Pre- and postoperative ranges of motion (*ROM*) and the degree of retroversion of the humeral stem of the megahead prosthesis

	Preoperative ROM				Postoperative ROM				
Patient	ER 1	IR 1	ER 2	IR 2	ER 1	IR 1	ER 2	IR 2	Retroversion
1	nm	nm	nm	nm	60	−10	80	80	40°
2	nm	nm	nm	nm	20	nm	nm	nm	40°
3	40	0	40	30	90	0	90	90	70°
4	40	90	80	na	10	nm	nm	nm	na
5	−20	0	nm	nm	−10	0	20	30	40°
6	na	na	na	na	80	−20	80	20	na
7	30	−70	10	10	30	−30	nm	nm	30°
8	na	na	na	na	na	na	na	na	70°
9	0	nm	nm	nm	20	−10	60	30	40°

*ER 1* external rotation in 0° anteflexion, 0° abduction and the elbow in 90° flexion; *IR 1* internal rotation in 90° anteflexion, 0° abduction with the elbow in 90° flexion; *ER 2* external rotation in 90° abduction with the elbow in 90° flexion; *IR 2* internal rotation in 90° abduction with the elbow in 90° flexion. *na* not available. *nm* not measurable

**Fig. 2:**
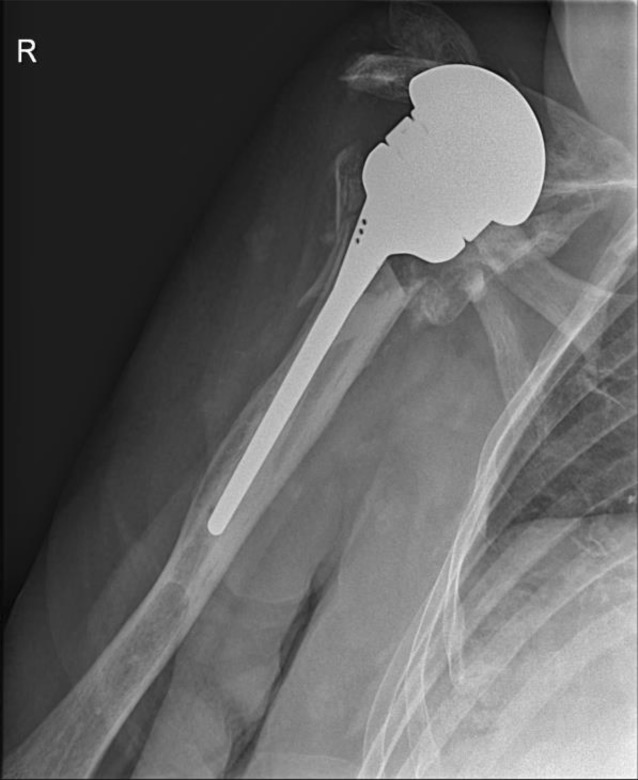
Patient 2: Anterosuperior migration of the megahead, 2 months after implantation

It must be noted that in 2 patients an anterosuperior escape of the megahead prosthesis was observed (Fig. 2).

Patient 7 had had multiple operations to treat infection of the initial RSA and was ultimately converted to a resection arthroplasty. Because of very poor function, a final attempt to implant a RSA was performed, though perioperatively a large bony insufficiency was found. The senior author decided perioperatively to implant a megahead prosthesis. Constant scores were poor (13/100) and did not improve postoperatively.

In 2 patients (patients 4 and 9) the megahead prosthesis was revised and ultimately converted to another RSA (type Delta XTEND, DePuy-Synthes). Patient 4 had a long history of infections of the shoulder prostheses: in 2003 an initial hemiprosthesis was revised to RSA because of infection with omnisensitive coagulase negative staphylococci (CNS). One year later, in 2004, the RSA was removed and converted to a megahead prosthesis because of CNS that was resistant to oxacilline, cotrimoxazol and gentamycin. This prosthesis remained functional till 2008 when another infection with CNS was detected: the megahead was removed and a hip spacer was placed. After 12 months, the spacer was finally converted to a RSA, which remains in place till current day, with improving ROM and a Constant score of 57. Perioperative cultures remained negative (Table 2).

**Table 2:** Postoperative evolution of patient 4 (female, 78 years at index procedure)

Patient 6	Megahead 3 months	Megahead 4 years	RSA 3 months	RSA 1 year
AF	150°	31–60°	121–150°	91–120°
AB	145°	31–60°	121–150°	91–120°
ER	na	nm	Hands in neck, elbows backwards	Hands on head, elbows backwards
IR	na	nm	Trochanter	Trochanter
Passive	na	0/nm/nm/nm	20/−30/60/60	10/−10/80/70

*AF* active anterior flexion; *AB* active abduction; *active ER* external rotation; *active IR* internal rotation; *Passive* ER 1/IR 1/ER 2/IR 2 as described above, *na* not available. *nm* not measurable

Patient 9 was revised because of very poor shoulder function 1 year after implantation of the megahead and increasing pain (VAS 8/10). The preoperative Constant score was 12 and improved to 52, only 3 months after conversion to RSA.

### Permanent spacers

**Fig. 3:**
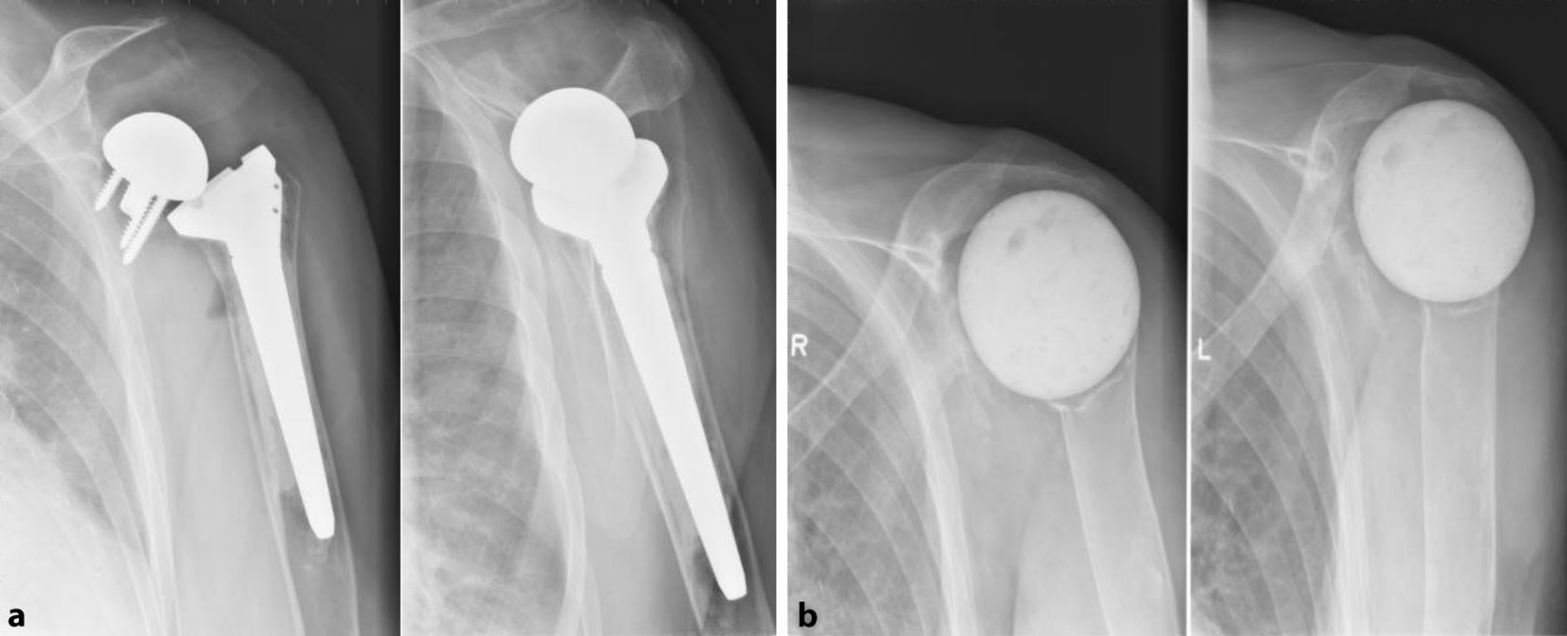
Patient 10: Loosening and infection of the RSA (*left*) and after implantation of a spherical cement spacer (*right*)

Seven spacers were implanted in 6 patients (4 males). We used the smallest size of commercially available hip spacers instead of shoulder spacers because of reimbursement reasons. Mean age at implantation of the spacer was 69.2 years. Two spacers remain in place till current day (patients 10 and 11). Patient 10 received a spherical spacer, made of gentamycin-impregnated cement, because of loosening due to persistent infection with CNS (Fig. 3). A hip spacer could not be used because of lateral and distal translation of the humerus.

**Fig. 4:**
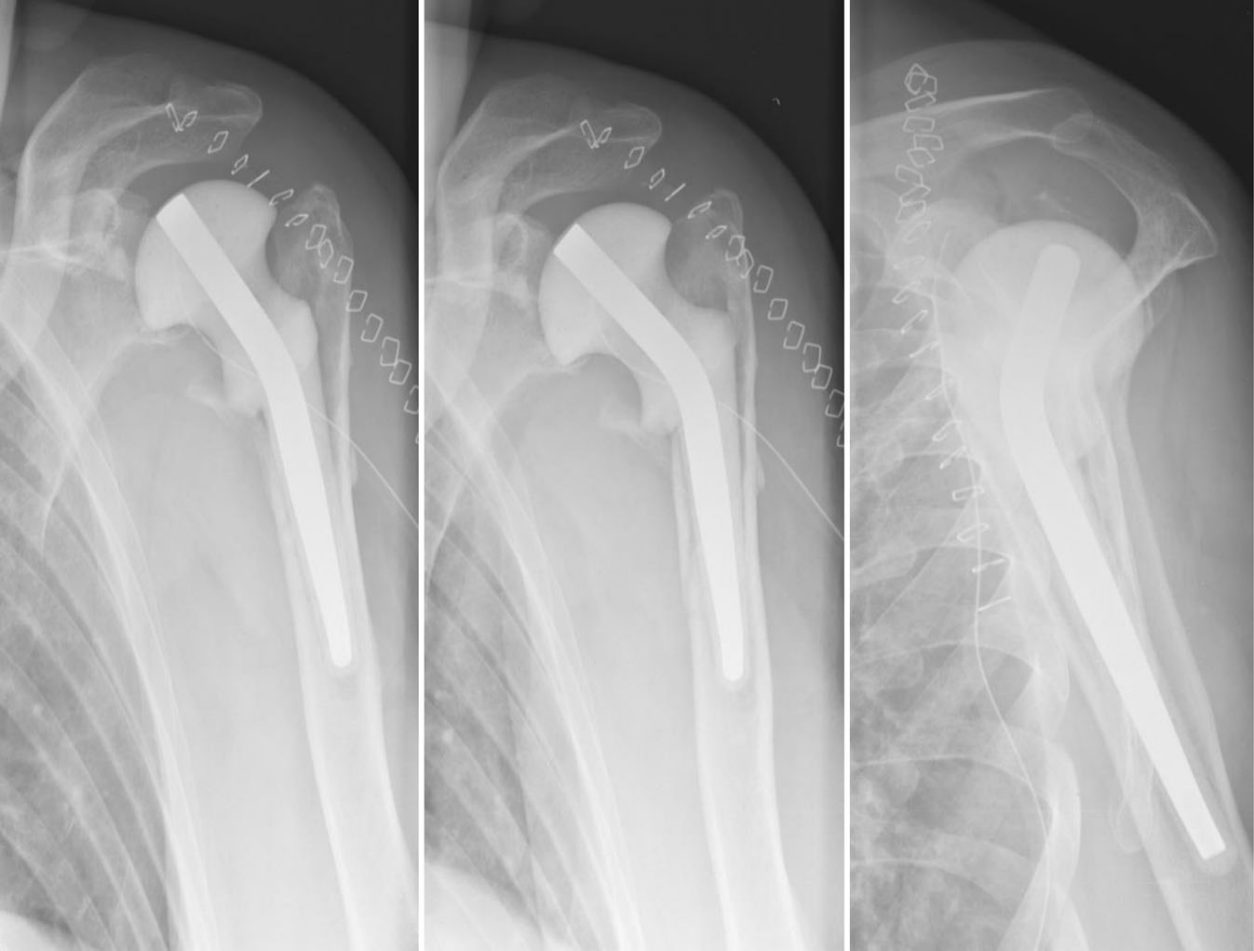
Patient 11: 1 day after conversion from RSA to a hip spacer

Patient 11 was implanted with a spacer, normally used for the hip, because of infection and dislocation of the RSA (Fig. 4). The preoperative Constant score was only 11, VAS 7/10 and passive mobility 10° in external rotation with the arm at the side. Six months after implantation of the hip spacer, the Constant score had improved to 37, with decrease of pain to 2.5/10, and an improvement of flexion and abduction from impossible to 60–90°. Passive mobility remained stable.

In the other 4 patients (patients 12–15), the spacer was removed after an average of 7.2 months because of pain, severe loss of function or recurrent infection. All patients could be treated with a RSA. Patient 12 had received 2 spacers: his first because of abscess formation of a primary RSA. After 8 months, a revision RSA was implanted. Unfortunately, reinfection occurred 13 months after revision, necessitating another spacer implantation. A final RSA was implanted 10 months after the second spacer.

### Resection arthroplasty

Five patients (patients 8, 16–19) were treated with resection arthroplasty when all other options failed. Mean age was 69.4 years; 3 out of 5 were males. Patient 1 was treated for infection of the right-sided RSA, which could not be controlled, and therefore the prosthesis was originally replaced by a hip spacer. Because of persistent infection, the spacer was ultimately removed and converted to a resection arthroplasty. Constant scores did not change and remained very poor (5/100); the level of pain was reported as high.

Patient 16 received an arthrodesis after two revisions of the initial RSA because of loosening and pain. Nevertheless, the pain was not relieved, and ultimately a resection arthroplasty was performed. Constant scores before and after surgery went from 7/100 to 10/100.

**Fig. 5:**
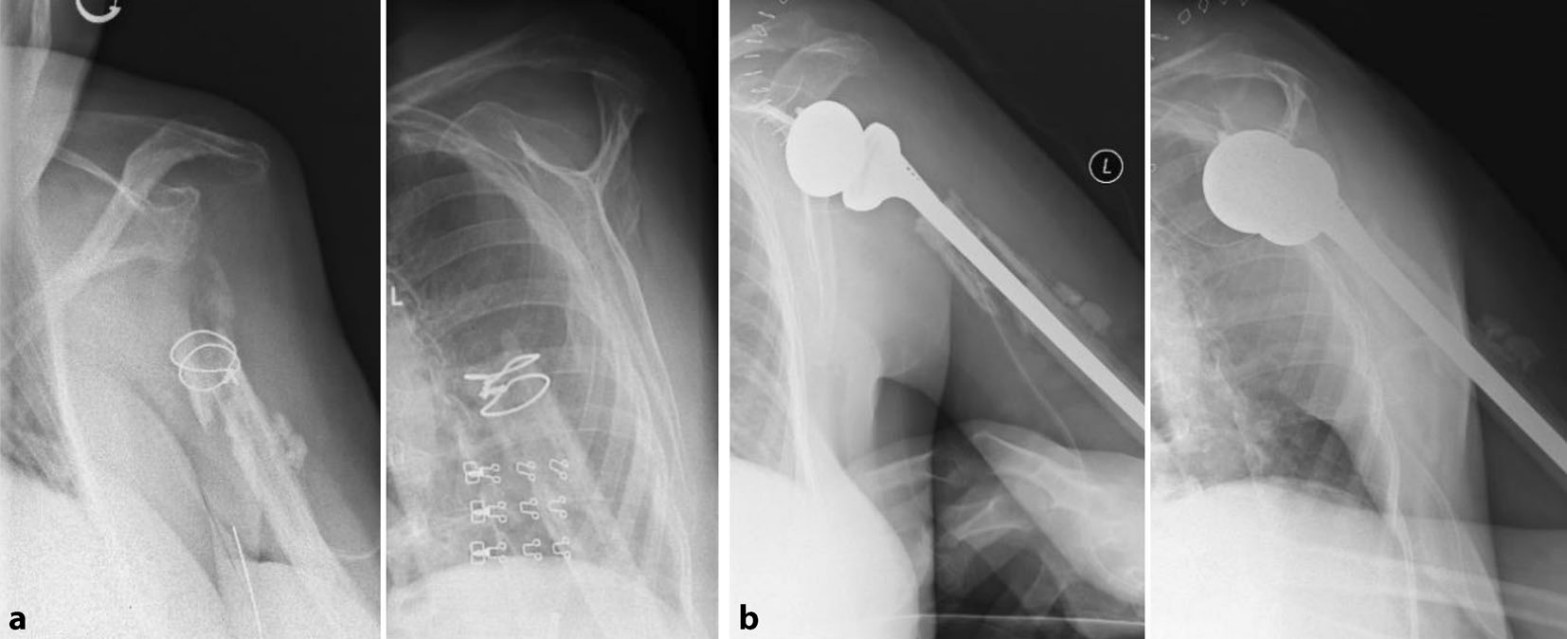
Patient 17: Status after resection arthroplasty (*left*) and 2 months after revision to RSA (*right*)

Three patients (patients 17–19) treated with resection arthroplasty were unsatisfied with their outcome, largely because of the pain. By mutual agreement, a final attempt to implant a RSA was performed (Fig. 5). Mean preoperative Constant score was 26.6/100. One year after surgery, all 3 RSA were in place, pain was relieved and mean Constant score had improved to 57.3. The clinical results are shown in Table 3.

**Table 3:** Comparison of 3 patients, initially treated with resection arthroplasty, who were converted to RSA. Constant scores (*CS*) before and 1 year after surgery

**Resection arthroplasty**						
**Patient**	**CS**	**Pain (/10)**	**AF (°)**	**AB (°)**	**IR**	**ER**
17	22	7.3	31–60	31–60	D12	NM
18	14	7	0–30	0–30	Trochanter	Hand to neck
19	44	6	61–90	61–90	L4	Hand on head
**1 year after conversion to RSA**						
**Patient**	**CS**	**Pain (/10)**	**AF (°)**	**AB (°)**	**IR**	**ER**
17	64	0	121–150	121–150	Sacro-iliacal joint	Hand over head
18	32	0	31–60	31–60	Buttock	NM
19	76	0	151–180	151–180	L4	Hand over head

*AF* active anterior flexion; *AB* active abduction; *ER* external rotation; *IR* internal rotation, *NM* not measurable

## Discussion

Reversed shoulder arthroplasty is a very successful treatment for a wide variety of shoulder pathologies, but along with the enormous increase in number of performed procedures, the number of complications and revisions has increased proportionally.

In our study, we found that the function after implantation of a megahead prosthesis is better than resection arthroplasty. The results however are inferior to RSA.

In our group of 20 shoulders in 19 patients with failing RSA who were initially treated with HA, spacer or RA because another RSA was thought to be impossible, it was ultimately possible to convert 9 to a RSA, as the surgical experience and technique of the senior author had increased, with satisfactory results concerning shoulder function and pain relief.

In case a revision is necessary but the preservation of a reversed implant is impossible, due to insufficient bone stock, infection or instability, a few procedures have been described (Table 4): hemiarthroplasty, spacer implantation, resection arthroplasty and specially devised implants.

**Table 4:** Review of current literature

Author + year	*N*	FU	Procedure	Indication (number of patients)	Postoperative outcome (mean ± SD)	Postoperative clinical scores (mean ± SD)	Note
Brodt 2010 [8]	1	>12 months	Hybrid spacer	Septic loosening of RSA	AF 40°; AB 30°; no pain	–	Full passive ROM
Debeer 2006 [20]	7	8.3 months	RA without spacer	Infection of TSA (3), osteosynthesis (1), arthrodesis (1), cuff repair (1), septic arthritis (1)	VAS 5.9/10 (=8.8/15)	CS 25.7; DASH 69.3	–
Gamradt 2012 [7]	6	26.5 months	HA after RSA	Dislocation (1), dislocation and infection (2), aseptic loosening (3)	AF 42.5°; ER 1.7°; VAS 2.42 ± 2.06	SST 3.17 ± 1.83; ASES 52.1 ± 9.32	Anterosuperior migration in 5
Ghijselings 2013 [17]	17	4.7 years	RA with spacer (4)	Infection of RSA, HA or TSA	VAS 6	CS 20.6; DASH 71.0; SST 1	7 patients of Verhelst et al. 12 of 17 patients included
Ghijselings 2013 [17]	17	4.7 years	RA without spacer (8)	Infection of RSA, HA or TSA	VAS 3.6	CS 27.8; DASH 46.9; SST 2.38	7 patients of Verhelst et al. 12 of 17 patients included
Glanzmann 2016 [12]	16	>24 months	HA after RSA	Glenoid loosening (11), infection (3), periprosthetic fracture	AF 45 ± 34°; ER 6 ± 10°; VAS 5	CS 25.2 ± 12.0; QuickDASH 63.0 ± 13.8; SPADI 36.7 ± 19.9	No difference Pre vs Postoperative, increase of pain in 5 patients
Muh 2013 [16]	26	41.8 months	RA	Failed TSA (6), HA (7), RSA (13)	AF 46.7 ± 29.1°; ER 8.9 ± 13.0°; VAS 3.2 ± 2.5	CS 27.3 ± 12.5; ASES 38.8 ± 7.0	No significant decrease of AF, ER or CS
Rispoli 2007 [18]	18	8.3 years	RA	Failed shoulder replacement (17) with infection (13), septic arthritis (1)	AF 70°, ER 31°, IR L5; VAS 4.5	ASES 36; SST 3.1	Significant decrease of pain (*p* < 0.001), although 5 patients continued to have moderate to severe pain. significant increase in AF (*p* = 0.003); no difference in ER or IR
Stevens 2015 [19]	7	2 years	RA	Uncontrolled pain in HA (2), RSA (3 in 2 patients), TSA (3)	AF 63°; ER 27°; VAS 3.3 ± 4.4	DASH 42.4 ± 23.6; ASES 49.75 ± 26.1; SST 4.9 ± 3.3	–
Themistocleous 2007 [13]	11	22 months	Antibiotic spacer	Septic arthritis after TSA (4), osteomyelitis (3), ORIF (2), cuff repair (2)	AB 75°; ER 25°	QuickDASH 37.5	No or mild pain; 9 patients were satisfied with spacer and wished no further surgery
Uri 2014 [21]	11	35 months	CAD/CAM after RSA	Glenoid failure + insufficient bone stock	AF 54 ± 17°; ER 21 ± 9°; VAS 2.3 ± 1.3	Oxford 33 ± 6, SSV 48 ± 17%	Maximal ROM of 60° around center of the prosthesis
Verhelst 2011 [14]	21	46.4 months	RA with/without spacer	Deep shoulder infection	AF 85.5 ± 43.1°; AB 78.1 ± 41.5°; ER 21 ± 16.2°; IR 15.6 ± 11.3°; VAS 2.6 ± 2.4	CS 40.4 ± 22.6; DASH 52.7 ± 22.2; SST 5.1/12 ± 3.5	No difference in CS with or without spacer; 5 patients with spacer were revised to HA or RSA

*AF* Anterior Flexion, *ASES* American Shoulder and Elbow Surgeons Shoulder Score, *AB* Abduction, *CS* Constant Score, *ER* External Rotation, *FU* follow-up, *IR* Internal Rotation, *(Quick) DASH* (Quick) Disabilities of the Arm, Shoulder and Hand Score, *ROM* Range Of Motion, *SPADI* Shoulder pain and Disability Index, *SST* Simple Shoulder Test, *VAS* Visual Analog Scale

### Hemiarthroplasty

Little literature has been published about the results of hemiarthroplasty as salvage after RSA. Zumstein et al. [4] described a cohort of 782 RSA, of which 9 were ultimately converted to HA. Farshad et al. [5] reported 3 hemiprostheses after glenoidal component complications and 1 spacer in a patient with suspected infection in a total of 67 cases needing additional intervention in a cohort of 441 RSA. In addition, Boileau described conversion of 2 patients from RSA to HA in 54 of the 825 RSA patients that required revision [11]. Gamradt et al. [7] reported about a series of 6 patients who underwent conversion from RSA to HA after an average of 9.2 months because of loosening of the glenoid (*n* = 3) and dislocation (*n* = 3, of whom 2 were infected). The patients who had an infection received a preformed antibiotic-loaded cement hemiarthroplasty. Shoulder function was reported as very poor, although pain was slight (2.42 ± 2.06).

A larger series was published by Glanzmann et al. [12]: 16 RSA patients, in whom the bone stock was insufficient for reimplantation of another RSA, underwent conversion to HA. The main indications were glenoid loosening (*n* = 11) and infection (*n* = 3). Comparison between pre- and postoperative shoulder function revealed no significant changes in pain, average anterior elevation, external or internal rotation. However, the authors note that in 5 patients the level of pain had increased after the hemiarthroplasty.

Our own results show 9 cases of conversion from RSA to a hemiprosthesis (megahead), of which 2 were ultimately reconverted to RSA. The latter can be explained because of the introduction of a revision baseplate (enlargement of the central peg to +10 mm or to +15 mm), the improvement of the surgical technique due to more surgical experience the bone stock appeared to be sufficient. In case that a stable fulcrum could be obtained we could give those patients reasonable shoulder function. This can be explained by the smooth lateral extension of the humeral head allowing gliding underneath the acromial arc. To obtain this fulcrum perioperative testing and an increased retroversion of the humeral implant together with a medialized glenoid (more medial than the coracoid process) are desirable.

### Spacer

Spacers are normally used in 2‑stage revisions as a temporary interposition, but in certain cases they can be a permanent solution if the patient is unable or unwilling to be subjected to another operation.

Brodt et al. [8] reported a case of septic loosening of a RSA that they had treated with a custom-made hybrid spacer, which contained gentamycin PMMA (polymethylmethacrylate). Although the aim was to reimplant an arthroplasty, the patient refused further surgery, as she was satisfied with the current result.

A larger study of 11 patients with septic shoulder arthritis treated with an antibiotic impregnated PMMA spacer in 30° retroversion was presented by Themistocleous et al. [13]. It must be noted that only 4 patients had an infected shoulder arthroplasty. At an average follow-up of 22 months, 9 of 11 patients were satisfied with the outcome and did not wish to undergo any further surgery.

Verhelst et al. [14] retrospectively investigated 21 patients after resection arthroplasty for persistent shoulder infection. They compared 10 patients who received a spacer (4 spherical, 6 long-stemmed) with 11 patients who did not. Using a spacer did not improve clinical or biochemical outcome, and higher complication and revision rates were found in the spacer group. In 5 patients with a spacer (1 spherical, 4 long-stemmed) severe glenoid bone loss was detected; these had a worse clinical result and an inferior CMS compared to the group with an intact glenoid. In another group of 5 patients treated with a spacer, a delayed prosthesis implantation was performed: one because of a periprosthetic fracture, 4 because of pain and limited function. Two patients received a hemiarthroplasty, 2 received a cuff tear arthropathy prosthesis, and 1 was treated with a Delta III RSA. No infection was found in the perioperative tissue samples.

It is our impression that stability can be enhanced by the tensioning of the deltoid and conjoined tendon, and lateralisation of the humeral shaft. Due to the improvement of the deltoid wrapping [15], a cement spacer of the hip is more suitable than a commercially available “anatomic” shoulder spacer in the different pathologies met during revision of RSA. A trial reduction and positioning in extreme retroversion might also benefit the construction of a stable fulcrum.

### Resection arthroplasty

Although a drastic treatment, resection arthroplasty has been described for shoulder infections of various origin. Several authors have evaluated this option in infected arthroplasties [16–20] and in most cases control of infection is achieved. Pain usually improves significantly, but some authors report worsening of pain in certain patients [18]. Also, functionality is poor and it must be noted that RA after failed RSA has worse functional outcomes than RA after TSA or HA. The Neer criteria for assessment of shoulder replacement (no/slight/intermittent moderate pain only with vigorous activity, active elevation to at least 90° and external rotation to at least 20°) are very seldom met and resection arthroplasty should therefore be considered unsuccessful. However patients are often satisfied because their pain has in most cases decreased, and their function before and after resection arthroplasty is usually comparable, or improving [18].

Most authors agree that resection arthroplasty is a valid therapeutic option in low demanding and highly painful shoulders after failed arthroplasty and that outcomes concerning function and pain not always can be predicted [16–20].

We advise this procedure only in cases of extreme weakness of the acromion/glenoid, in case of deltoid/conjoined tendon dysfunction (due to axillary nerve lesion) or due to deltoid dehiscence problems where it can serve as a salvage procedure because it is always better than an amputation of the upper limb. In our own experience however, we saw that reconversion to RSA was often possible (cf. Table 3), resulting in pain relief and marked improvement of function.

### CAD/CAM

Uri et al. [21] described a hip-inspired implant for revision of failed RSA with severe glenoid bone loss: their cohort of 11 failing-RSA patients underwent revision with the CAD/CAM (computer-assisted design/computer-assisted manufacture) shoulder (Stanmore Implants, Elstree, UK), which consisted of a large uncemented titanium glenoid shell mated to a cobalt–chrome tapered humeral stem. At a mean follow-up of 35 months, mean active anterior elevation and external rotation improved significantly, contrary to internal rotation. Pain was eased, especially during activity. The Oxford shoulder score decreased from 50 ± 4 to 33 ± 6, and the subjective shoulder value increased from 17 ± 11% to 48 ± 17 %.

We do not have any experience with this type of surgery but the early results seem to be promising.

### Glenohumeral arthrodesis

Rühmann et al. [22] reported 2 arthrodeses after prosthesis removal in their group of 43 arthrodeses. They described difficult stable fixation because of reduced bony conditions, and bone grafting was performed in 1 patient.

In the report of Scalise and Iannotti [10], stable fusion was reached in 5 of 7 arthrodesis patients with severe glenohumeral bone loss and insufficiency of the rotator cuff and deltoid muscles after TSA. Additional bone-grafting procedures had to be performed in 4 patients, resulting in 2 unions.

We do not have any experience either and consider this treatment only in case of sufficient bone stock and failure of the resection arthroplasty.

### Megahead CTA prosthesis

This study is to our knowledge the first to report on the results of the megahead CTA prosthesis in failure of RSA. The advantages of this prosthesis are the smooth lateral extension allowing for good gliding underneath the acromial arc. In case of a disruption of this arc it is advisable to look for bony stability to oppose the ascending force of the deltoid. This can be enhanced by implanting the prosthesis in more retroversion and by the medialisation of the centre of rotation which is often the case in severe glenoid erosion. In case of failure of the bony arc of the acromion/coracoid process we advise not to implant this type of prosthesis.

This study has also several limitations. First, the number of patients treated with a megahead prosthesis at our institution is relatively small, despite being a tertiary referral centre. Second, there was the retrospective design and therefore, not all data were available for all patients. Constant scores were not always completed or were lost in administration. Due to the incomplete data, no statistical analysis was possible.

On the other hand we consider it useful to report on these numbers so that future review articles can provide us better information concerning this rare surgery, which is becoming increasingly common.

## Conclusion

In our case series study a hemiarthroplasty can be performed in case of revision. However the results are inferior to another RSA. Resection arthroplasty can be considered to relieve pain if the HA should fail, sometimes but not always at the cost of shoulder function. Arthrodesis should only be performed if the only alternative is amputation. Furthermore it must be noted that in almost half of the salvage procedures (9 of 19 patients) we performed (HA, spacer, RA), it was possible to reconvert the salvage to a RSA because of improvement of technique and growing surgical experience.
